# Effect of prior cancer on survival of hepatocellular carcinoma: implications for clinical trial eligibility criteria

**DOI:** 10.1186/s12885-021-07870-0

**Published:** 2021-02-09

**Authors:** Xuqi Sun, Lingling Li, Li Xu, Zhongguo Zhou, Jinbin Chen, Juncheng Wang, Yaojun Zhang, Dandan Hu, Minshan Chen

**Affiliations:** 1 State Key Laboratory of Oncology in South China, Collaborative Innovation Center for Cancer Medicine, Sun Yat-Sen University Cancer Center, Guangzhou, 510060 China; 2 Department of Liver Surgery, Sun Yat-Sen University Cancer Center, Guangzhou, 510060 China; 3 Zhongshan School of Medicine, Sun Yat-Sen University, Guangzhou, 510060 China; 4 Department of Liver Surgery, Sun Yat-Sen University Cancer Center, Dongfeng East Road 651, Guangzhou, Guangdong 510000 People’s Republic of China

**Keywords:** Hepatocellular carcinoma, Prior cancer, Survival, Clinical trials

## Abstract

**Background:**

Patients with cancer history are usually excluded from hepatocellular carcinoma (HCC) clinical trials. However, whether previous malignancy affects the oncological outcomes of HCC patients has not been fully assessed. This study aimed to evaluate whether prior cancer compromised the survival of HCC patients.

**Methods:**

Patients with HCC were extracted from the Surveillance, Epidemiology, and End Results database between 2004 and 2015, and then they were classified into groups with and without prior cancer. The Kaplan-Meier and multivariate Cox regression analysis were adopted to evaluate whether prior cancer impacted clinical outcomes after propensity score matching (PSM) adjusting baseline differences. Validation was performed in the cohort from our institution.

**Results:**

We identified 2642 HCC patients with prior cancer. After PSM, the median overall survival (OS) time were 14.5 and 12.0 months respectively for groups with and without prior cancer. Prior cancer did not compromise prognosis in patients with HCC (*p* = 0.49). The same tendency was found in subgroups stratified by tumor stages and cancer interval period: OS was similar between groups with and without prior cancer (both *p* values> 0.1). In the multivariate Cox regression model, prior cancer did not adversely impact patients’ survival (HR: 1.024; 95% CI: 0.961–1.092). In the validation cohort from our institution, prior cancer had no significant association with worse outcomes (*p* = 0.48).

**Conclusion:**

For HCC patients, prior cancer did not compromise their survival, regardless of tumor stage and cancer interval period. Exclusion criteria for HCC clinical trials could be reconsidered.

**Supplementary Information:**

The online version contains supplementary material available at 10.1186/s12885-021-07870-0.

## Background

Hepatocellular carcinoma (HCC) ranks as the sixth most common malignancy worldwide [1]. While patients with early-stage HCC can achieve promising survival after curative treatments, the 5-year survival is only 18% among those at advanced stage, who account for 80% of the total HCC population [2]. A complete remission of lesions can hardly be achieved and the cancer has generally evolved into a systemic disease. Treatment modalities are very limited in these settings, making the therapeutic strategy extremely demanding.

For decades, clinicians have been dedicated to different clinical trials to develop better treatments for the appropriate patients, especially for late-stage cancer patients. Clinical trials improve management and treatments for cancer patients. Regarding targeted therapy for HCC alone, clinical trials have proved sorafenib and lenvatinib as the first-line treatments, regorafenib, cabozantinib and pembrolizumab as the second-line options that can prolong patients’ survival [3, 4]. Evidence-based medicine are providing us with better practice in the management of patients, and ongoing clinical trials contribute to future guidelines of standard therapies. It is mandatory to adopt the best current therapy in the control group and offer financial allowance in most cases, aiming to guarantee the benefits of enrolled patients.

Albeit the fact that clinical trials provide various potential positive effects for cancer patients, only 2–4% of this population are recruited into trials in the US [5, 6]. This low accrual rates can compromise the efficiency and generalizability of trials. Stringent eligibility criteria seriously hamper the trial enrollment, which usually excludes patients with prior malignancy [7]. Cancer history is an exclusion criterion in approximately 80% of lung cancer clinical trials due to the low-grade evidence that prior cancer can compromise patients’ survival [6]. Zhou et al. have suggested not all prior malignancy influence treatment outcomes of subsequent cancers [8]. Given that cancer survivors have increased nearly four-fold over the past three decades, excluding patients with prior cancer will further limit the generalizability of trials [9]. We aimed to evaluate how prior cancer and intervals of cancers affect prognosis of HCC patients with the data from the Surveillance, Epidemiology, and End Results (SEER) database and our institution, which represent different population and etiology of HCC.

## Methods

### Patients and data collection

Patients with HCC were identified in the SEER database from 2004 to 2015, which was composed of 17 population-based cancer registries and covered around 30% of the total population in the United States (US) [10]. The exclusion criteria included: 1) diagnosed with HCC at age ≤ 18 years old; 2) die within 1 month after diagnosis; 3) without active follow-up. For patients with prior cancer before HCC, we only included those having HCC as the second primary cancer considering history of multiple prior cancers may indicate unfavorable germline mutations and interfere with outcomes.

To validate our findings from the SEER database, we reviewed HCC patients in our institution from 2009 to 2017 and enrolled 53 patients with prior cancer. We also randomly enrolled 53 patients only with primary HCC at the same period. We collected demographic and clinicopathological characteristics of patients from the SEER database and our institution, including gender, age at diagnosis with HCC, pathologic grade, tumor stage, treatment modality and prior cancer history.

The eligible patients were classified based on prior cancer history. Cancer interval was defined by the diagnosis interval between two cancers. The overall survival (OS), as the primary outcome, was calculated from the date of HCC diagnosis to the date of death or the latest following. Cancer-specific survival (CSS) was measured between diagnosis to HCC-related death or the latest follow-up. Progression-free survival (PFS) was evaluated in validation cohort, which was defined from HCC diagnosis to recurrences or tumor progression.

### Statistical analysis

The demographic and clinicopathological characteristics were assessed with chi-square test and t-test between groups with and without prior cancer. The 1:1 propensity score matching (PSM) was adopted for balancing confounding factors between subgroups [11]. Multivariate Cox regression analysis were adopted to assess whether prior cancer had adverse effects on patients’ outcomes. Kaplan-Meier (K-M) method with log-rank test was adopted for comparing OS, CSS and PFS. The basic characteristics were entered as covariates including gender, race, age, tumor size and stage, pathologic grade and treatment modality. *P* value < 0.05 was significantly different. Data analyses were performed with R version 3.5.3.

## Results

### Patient characteristics before and after PSM

In total, 47,431 eligible patients with HCC were identified, and 2642 ones had previous cancer history. Prostate (31.1%), genitourinary and gynecologic (17.2%) and gastrointestinal (15.3%) were the most common prior cancers. Patients with prior malignancy tended to be older and at earlier HCC stages. In this study, the curative treatments consisted of liver transplantation, resection and ablation. The proportion of curative treatments for HCC was similar between the two groups with and without prior cancers. The proportion of systemic therapy and radiotherapy was slightly higher in HCC patients with prior cancer. The radiotherapy patients receiving included external radiation and brachytherapy. All covariates were balanced between the two groups after PSM. Detailed information was presented in Table 1. The median time of cancer interval period was 47 months.

**Table 1:** Baseline characteristics of the patients with HCC from SEER database

Patient characteristics	Raw cohorts			PSM cohorts		
	No prior cancer (*n* = 44,789)	With prior cancer (*n* = 2642)	*P* value	No prior cancer (*n* = 2642)	With prior cancer (*n* = 2642)	*P* value
**Age** (years)	62.57 ± 10.80	68.95 ± 9.77	< 0.001	69.01 ± 9.84	68.95 ± 9.77	0.843
**Sex**			0.852			0.472
Female	10,200 (22.8)	597 (22.6)		620 (23.5)	597 (22.6)	
Male	34,589 (77.2)	2045 (77.4)		2022 (76.5)	2045 (77.4)	
**Race**			< 0.001			0.141
White	30,250 (67.5)	1903 (72.0)		1951 (73.8)	1903 (72.0)	
Black	5843 (13.0)	386 (14.6)		337 (12.8)	386 (14.6)	
Others/unknown	8717 (19.4)	353 (13.4)		354 (13.4)	353 (13.4)	
**Tumor size** (mm)			0.102			0.761
0 < x ≤ 20	5733 (12.8)	297 (11.2)		290 (11.0)	297 (11.2)	
20 < x ≤ 50	17,424 (38.9)	1061 (40.2)		1039 (39.3)	1061 (40.2)	
50 < x ≤ 100	10,166 (22.7)	628 (23.8)		616 (23.3)	628 (23.8)	
x > 100	4419 (9.9)	257 (9.7)		265 (10.0)	257 (9.7)	
Unknown	7047 (15.7)	399 (15.1)		432 (16.4)	399 (15.1)	
**AJCC stage**			< 0.001			0.683
I	16,421 (36.6)	1089 (41.2)		1110 (41.6)	1089 (41.2)	
II	8861 (19.8)	500 (18.9)		507 (19.2)	500 (18.9)	
III	8483 (18.9)	462 (17.4)		481 (18.2)	460 (17.4)	
IV	4625 (10.3)	228 (8.6)		223 (8.4)	228 (8.6)	
Unknown	6399 (14.3)	365 (13.8)		331 (12.5)	365 (13.8)	
**Pathological grade**			< 0.001			0.196
Well differentiation	5487 (12.3)	450 (17.0)		453 (17.1)	450 (17.0)	
Moderately differentiation	7452 (16.6)	554 (21.0)		517 (19.6)	554 (21.0)	
Poorly differentiation / undifferentiation	3381 (7.5)	222 (8.4)		193 (7.3)	222 (8.4)	
Unknown	28,469 (63.6)	1416 (53.6)		1479 (56.0)	1416 (53.6)	
**Curative treatment**			0.902			0.952
No/unknown	31,473 (70.3)	1853 (70.1)		1856 (70.2)	1853 (70.1)	
Yes	13,316 (29.8)	789 (29.9)		786 (29.8)	789 (29.9)	
**Radiotherapy**			0.004			0.097
No/unknown	41,244 (92.1)	2391 (90.5)		2426 (91.8)	2391 (90.5)	
Yes	3545 (7.9)	251 (9.5)		216 (8.2)	251 (9.5)	
**Systemic therapy**			0.068			0.088
No/unknown	25,853 (57.7)	1478 (55.9)		1539 (58.3)	1478 (55.9)	
Yes	18,936 (42.3)	1164 (44.1)		1103 (41.7)	1164 (44.1)	

### Patient survival

**Fig. 1:**
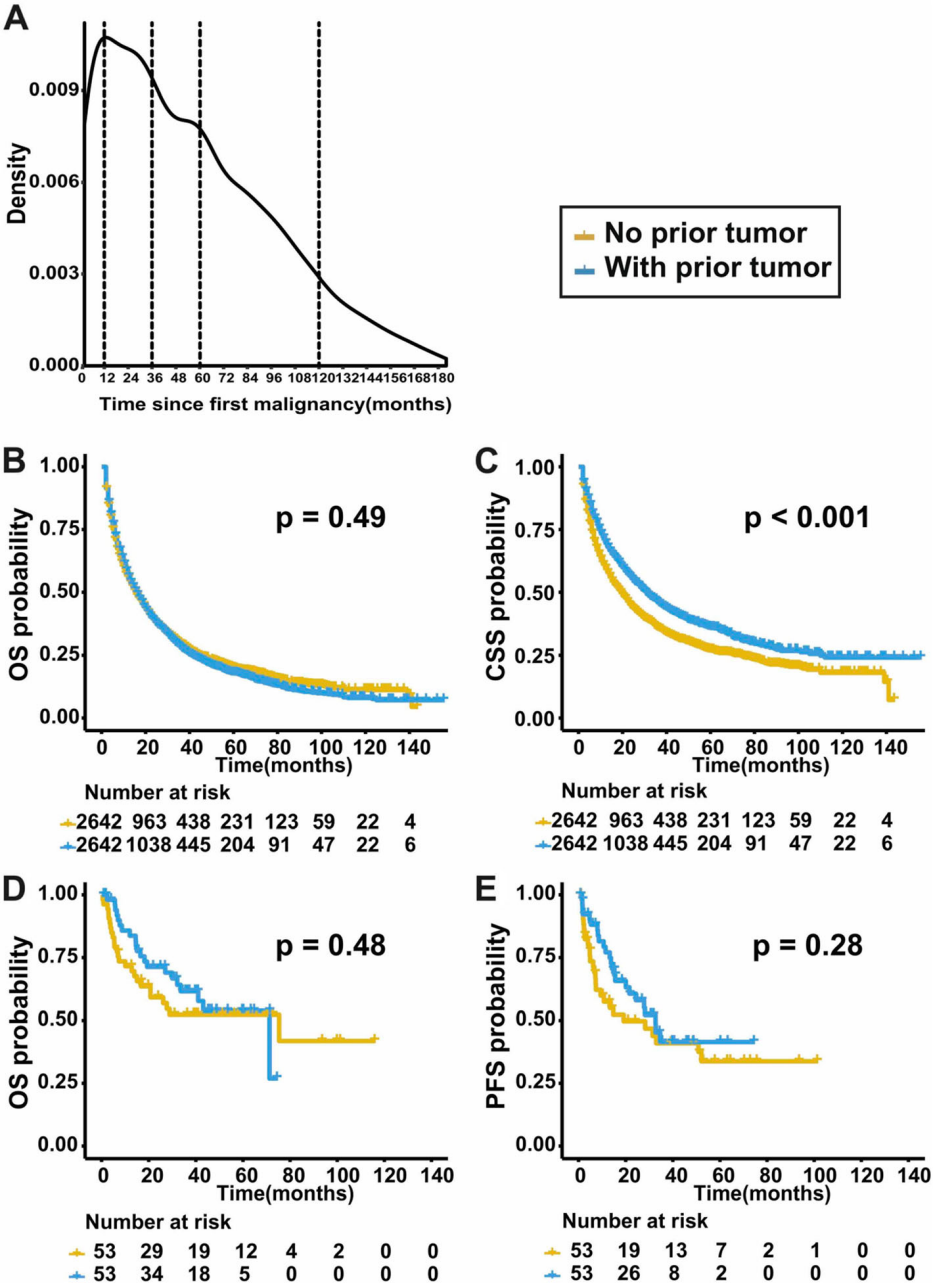
The outcomes of patients from the SEER database and our institution. **a** The probability density curve of HCC as second primary malignancy showed that subsequent cancer centered in the first 3 years after prior cancer. The OS (**b**) and CSS (**c**) of patients with and without prior cancer in PSM cohort from the SEER database. The OS (**d**) and PFS (**e**) of patients with and without prior cancer from our institution

**Fig. 2:**
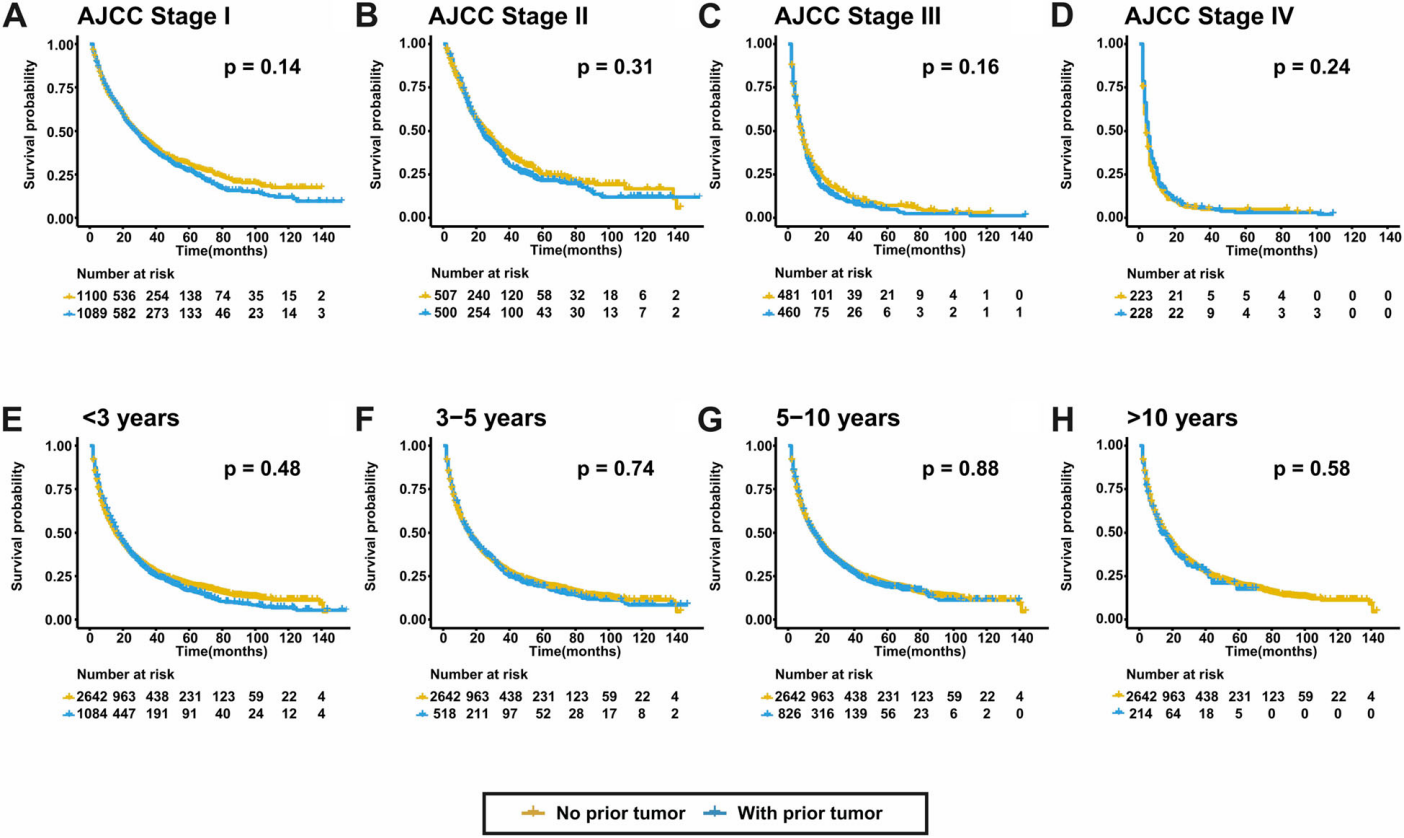
The OS of subgroups stratified by tumor stages and cancer interval period. Patients with prior cancer had similar OS to those with no prior cancer

**Fig. 3:**
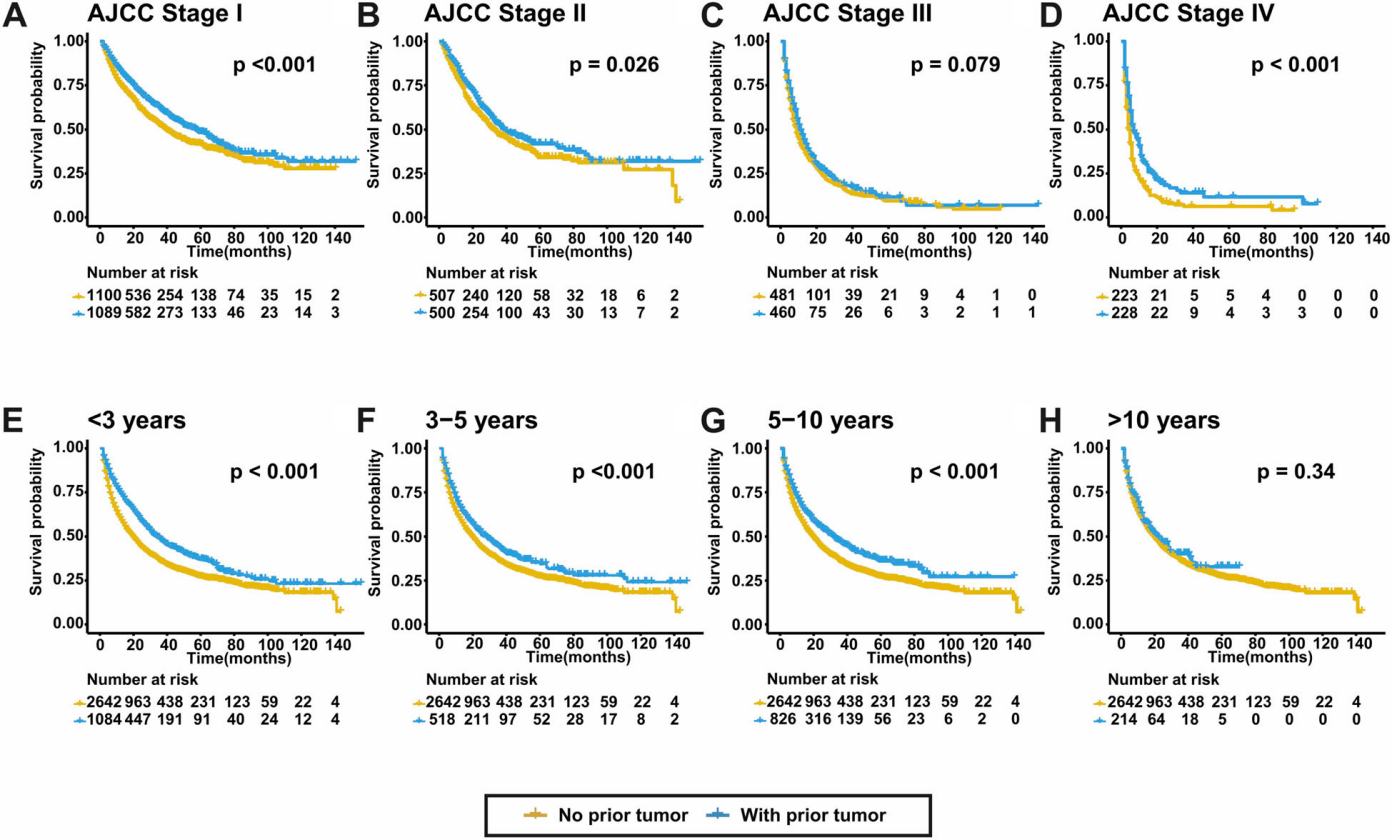
The CSS of subgroups stratified by tumor stages and cancer interval period. Prior cancer did not compromise patients’ CSS

For patients with prior cancer, a subsequent primary HCC centered in the first 3 years according to the probability density plot (Fig. 1a). 1602 (60.64%) patients were diagnosed with HCC within the first 5 years. The median survival time after HCC diagnosis were 14.5 and 12.0 months for groups with and without prior malignancy. The OS was comparable between the two groups (*p* = 0.49) (Fig. 1b). Figure 2 depicts the K-M survival curves between groups with and without prior malignancy stratified by tumor stages and cancer interval time. Prior cancer did not compromise OS in all subgroups. For CSS, prior cancer had a favorable effect on HCC (*p* < 0.001) (Fig. 1c). The similar tendency was found in subgroups stratified by tumor stages and cancer interval period, which was shown in Fig. 3.

**Fig. 4:**
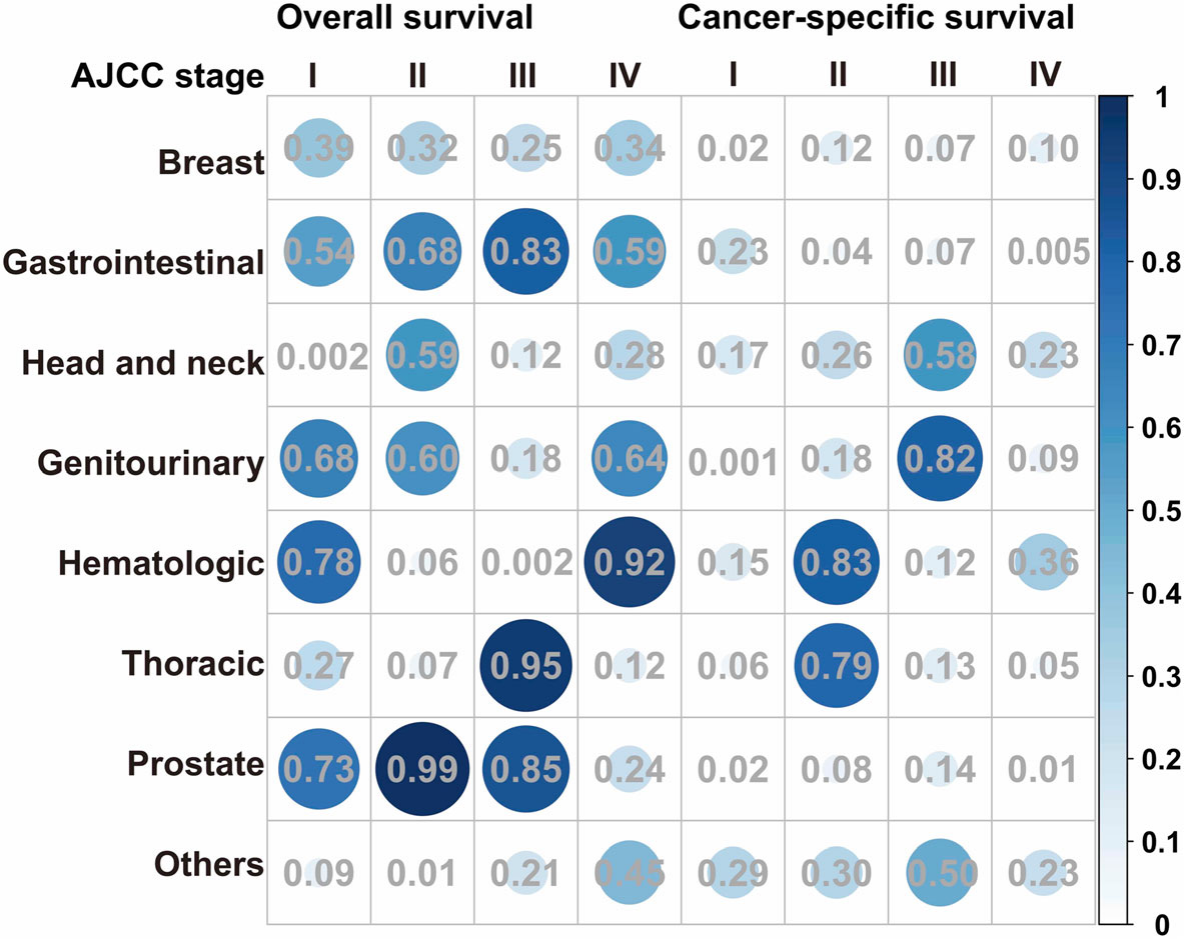
Subgroup analysis of different prior cancers impact on the OS and CSS stratified by stage of HCC in PSM cohort. Except for head and neck and hematologic malignancy adversely affecting OS of stage I and III HCCs, patients with prior cancer had non-inferior OS and CSS than those without prior cancer

After evaluating the effect of each specific prior cancer on different AJCC stage HCCs, most types of prior cancer did not significantly affect OS in each stage. Head and neck, and hematologic cancers adversely affected the OS of stage I and III HCCs respectively but did not relate with CSS in general population of HCC. Patients with some types of prior cancer had better CSS than those without in specific AJCC stage including breast, gastrointestinal, genitourinary and prostate cancers. Summarily, patients with other prior malignancies had non-inferior CSS than those without. Detailed data were shown in Fig. 4.

In the adjusted cox regression analysis after PSM, the group with prior cancer had comparable OS but superior CSS in comparison with that without (95% confidence interval of HR: 0.96–1.09 and 0.68–0.79, respectively). Detailed information is shown in Table 2.

**Table 2:** Multivariate Cox regression analyses on patients with HCC from SEER database

Multivariate Cox regression analyses	Overall survival		Cancer-specific survival	
	HR^a^ (95%CI)	*P* value	HR^a^ (95%CI)	*P* value
**Age** (years)	1.013 (1.010–1.017)	< 0.001	1.015 (1.011–1.020)	< 0.001
**Sex**				
Female	Reference		Reference	
Male	0.975 (0.903–1.054)	0.526	0.950 (0.867–1.040)	0.265
**Race**				
White	Reference		Reference	
Black	1.040 (0.947–1.142)	0.412	1.002 (0.896–1.121)	0.976
Others/unknown	0.741 (0.671–0.817)	< 0.001	0.752 (0.670–0.843)	< 0.001
**Tumor size** (mm)				
0 < x ≤ 20	Reference		Reference	
20 < x ≤ 50	1.300 (1.149–1.471)	< 0.001	1.396 (1.193–1.633)	< 0.001
50 < x ≤ 100	1.608 (1.401–1.847)	< 0.001	1.860 (1.567–2.207)	< 0.001
x > 100	2.312 (1.982–2.698)	< 0.001	2.797 (2.321–3.371)	< 0.001
Unknown	2.008 (1.733–2.326)	< 0.001	2.253 (1.878–2.701)	< 0.001
**AJCC stage**				
I	Reference		Reference	
II	1.163 (1.057–1.279)	0.002	1.250 (1.113–1.404)	< 0.001
III	1.861 (1.685–2.056)	< 0.001	2.088 (1.862–2.341)	< 0.001
IV	2.517 (2.235–2.834)	< 0.001	2.886 (2.519–3.306)	< 0.001
Unknown	1.407 (1.257–1.576)	< 0.001	1.534 (1.341–1.755)	< 0.001
**Pathological Grade**				
Well differentiation	Reference		Reference	
Moderately differentiation	1.116 (1.001–1.245)	0.048	1.199 (1.054–1.363)	0.006
Poorly differentiation / undifferentiation	1.569 (1.372–1.794)	< 0.001	1.668 (1.428–1.949)	< 0.001
Unknown	1.277 (1.166–1.398)	< 0.001	1.266 (1.137–1.410)	< 0.001
**Curative treatment**				
No/unknown	Reference		Reference	
Yes	0.429 (0.394–0.467)	< 0.001	0.401 (0.362–0.445)	< 0.001
**Radiotherapy**				
No/unknown	Reference		Reference	
Yes	0.934 (0.836–1.044)	0.231	0.960 (0.843–1.093)	0.534
**Systemic therapy**				
No/unknown	Reference		Reference	
Yes	0.904 (0.848–0.965)	0.002	0.976 (0.905–1.053)	0.534
**Prior cancer history**				
No	Reference		Reference	
Yes	1.024 (0.961–1.092)	0.462	0.736 (0.682–0.793)	< 0.001

^a^*HR* Hazard ratio

### Validating the effects of prior cancer

With the same criteria adopted in the SEER database, we identified 106 patients from our center. Patients with prior cancer tended to be older and at earlier stage than those without prior cancer (supplement Table 1). In cox regression analysis, prior cancer did not significantly corelate with PFS or OS (*p* = 0.303 and 0.996) (supplement Table 2). The median PFS times for groups with or without prior malignancy were 22.32 and 23.16 months, and the median OS times were 31.26 and 33.93 months, respectively. As in the SEER cohort, OS and PFS were similar in groups with and without prior cancer (*p* = 0.48 and 0.28), which was shown in Fig. 1d and e.

## Discussion

In current study, we found that HCC patients with prior cancer did not have inferior clinical outcomes than those without. The interval between two types of cancers had no significant effect on the survival data of HCC. There are several reasons for eliminating patients with prior malignancy from clinical trials. The predominant one is the long-holding assumption that antecedent malignancy can affect oncological outcomes [12]. Prior cancer can make patients less responsive to treatments for the newly onset malignancy. Some sponsors may thus forbid investigators to enroll such patients, though US Food and Drug Administration (FDA) have not recommended exclusion of prior cancer patients in the study design [13].

Few high-grade evidence support any side of the argument currently, and excluding patients with prior cancer limits external validity of clinical trials. Though proposals have been calling to simplify clinical trial eligibility, accrual criteria are increasingly stringent. Patients with comorbidities or antecedent cancer are commonly excluded from trials, which reflexively hinder efforts to increase study participation. In a randomized phase III HCC study, 458 (27.5%) patients were excluded for not meeting the eligibility criteria [14]. Compared to other comorbidities, history of prior cancers excluded over twice of patients in recent lung cancer studies [15]. The increasing cancer survivors will expand the adverse influence of this issue.

Oncological outcomes are comparable in both groups from the SEER database and validation of our own data. In the adjusted survival analysis, patients with prior malignancy had comparable OS but superior CSS comparing to those without. After specifically evaluating how different prior cancers affect OS and CSS of different AJCC stage HCCs, only head-and-neck and hematologic cancers adversely related with OS in specific stages, but no significant correlation was found in CSS. It may be counterintuitive that prior cancer was a protective factor in CSS. Ya et al. have reported similar results in nasopharyngeal carcinoma (NPC), and they inferred that prior cancer can cause more non-NPC deaths [16]. Apart from that, potential explanations include favorable tumor biology and better compliance to treatments [17]. Lead-time bias can also contribute to favorable CSS in patients with prior cancer since this cohort tend to receive more intensive surveillance than those without prior cancer and thus lesions are detected more promptly [13]. Bian et al. found that HCC patients with prior cancers could achieve comparable all-cause or cancer-specific survival comparing with those without [18]. However, confounding variables were not well balanced in their study. Additionally, though HCC can be diagnosed by imaging modality, they only included patients with microscopically confirmed HCC, which may lead to the bias of enrolling patients receiving hepatectomy for early-stage HCC [2]. Only 11.8% of their cohort were at advanced stage, while the portion was more than 25% in our research. Clinical trials are relatively more urgent and universal for late-stage cancer patients than those at early stage since curative therapy including resection and ablation can achieve satisfactory outcomes.

Till now, there are insufficient data on whether patients with prior cancer should be eliminated from clinical trials. Zhou et al. have reported the varying impact of prior cancers on subsequent malignancy, and the survival of HCC was not significantly influenced by prior cancer history [8]. Similarly, several studies have demonstrated that prior cancer did not compromise survival in several cancers including nasopharyngeal, lung and pancreatic cancers [16, 19, 20]. The National Cancer Institute Cancer Therapy Evaluation Program suggested that patients with curative treatments for previous malignancy and no recurrence for 5 years are eligible for clinical trials for subsequent cancers [21]. Our subgroup analysis stratified by cancer interval period showed the influence of prior malignancy was independent of cancer interval, thus HCC patients with history of cancer can be enrolled into clinical trials regardless of the interval period.

Prior cancer therapy may compromise patients’ tolerance for experimental treatments, though this study could not evaluate therapy toxicities. Blood tests and organ function can solve this concern. Prior treatment but not cancer can be regarded as exclusion criteria since curative surgery is less likely to affect patients’ physical function compared with systemic therapy, and this may exclude much fewer participators [22]. Stratified analyses or Cox model can be adopted to better adjust potential confounding effects by prior cancer.

This study has several limitations except for its retrospective nature. First, it is lack of detailed clinical characteristics of patients identified from the SEER database. Though we have adopted PSM to balance observed covariates, hidden bias from unobserved factors may potentially confound our results. Second, there is a paucity of surveillance and diagnosis, so lead-time bias from intensive follow-up in patients with prior cancer can hardly be well assessed. To overcome this potential confounding, we’ve balanced tumor size and stage between subgroups to ensure patients diagnosed at comparable situation. Third, the SEER database consists of patients only from the US, whether our results are applicable in other populations remains uncertain. We have validated our findings with data from our institution, the largest cancer center in southern China, and similar results were obtained. Forth, some subgroups consisted of limited numbers of patients in the Fig. 4, especially for the subgroups in stage IV. These limited sample sizes might weaken the statistic effectiveness, so multicenter studies are needed to further verify these results.

## Conclusion

For patients with HCC, prior cancer does not compromise patients’ clinical outcomes, regardless of tumor stage and cancer interval period. These results indicated that simplified eligibility criteria can potentially be adopted in HCC clinical trials, though well-designed prospective clinical trials are called to further validate these findings.

## Supplementary Information

**Additional file 1: Supplement Table 1.** Baseline characteristics of the patients with HCC diagnosed in 2009–2017 from Our Cancer Center.

**Additional file 2: Supplement Table 2.** Independent factors for the survival of HCC patients in 2009–2017 from Our Cancer Center.

## Data Availability

The datasets used during the current study are available from the corresponding author on reasonable request.

## Abbreviations

HCC: Hepatocellular carcinoma
SEER: the Surveillance, Epidemiology, and End Results
US: the United States
OS: Overall survival
CSS: Cancer-specific survival
PFS: Progression-free survival
PSM: Propensity score matching
K-M: Kaplan-Meier
FDA: US Food and Drug Administration
NPC: Nasopharyngeal carcinoma
