# Supplementary material for: Effect of prior cancer on survival of hepatocellular carcinoma: implications for clinical trial eligibility criteria
Source: BMC Cancer. 2021 Feb 9;21:147. doi: 10.1186/s12885-021-07870-0 (PMC7871582; doi:10.1186/s12885-021-07870-0)
Supplement: Supplementary file 1 — Additional file 1: Supplement Table 1. Baseline characteristics of the patients with HCC diagnosed in 2009–2017 from Our Cancer Center. [file 12885_2021_7870_MOESM1_ESM.docx]

**Supplement Table 1.** Baseline characteristics of the patients with HCC diagnosed in 2009-2017 from Our Cancer Center.

| **Patient Characteristics** | **No prior cancer** | **With prior cancer** | **P value** |
| --- | --- | --- | --- |
|  | **n=53** | **n=53** |  |
| **Age** (years) | 53.42±12.52 | 63.47±9.53 | <0.001 |
| **Sex** | | | 0.597 |
| Female | 7 (13.2) | 10 (18.9) |  |
| Male | 46 (86.8) | 43 (81.1) |  |
| **HBV infection** | | | 0.070 |
| No | 5(9.4) | 13(24.5) |  |
| Yes | 48 (90.6) | 40 (75.5) |  |
| **Cirrhosis** | | | 0.694 |
| No | 32(60.4) | 29(54.7) |  |
| Yes | 21 (39.6) | 24 (45.3) |  |
| **Tumor size** (mm) | 66.08±39.56 | 52.74±34.45 | 0.067 |
| **Tumor number** | | | 0.409 |
| Solitary | 33 (62.3) | 38(71.7) |  |
| Multiple | 20 (37.7) | 15 (28.3) |  |
| **AJCC stage** | | | 0.016 |
| I | 27 (50.9) | 36 (67.9) |  |
| II | 4 (7.5) | 8 (15.1) |  |
| III | 20 (37.7) | 6 (11.3) |  |
| IV | 2 (3.8) | 3 (5.7) |  |
| **BCLC stage** | | | 0.050 |
| 0/A | 29 (54.7) | 38 (71.7) |  |
| B | 11 (20.8) | 11 (20.8) |  |
| C | 13 (24.5) | 4 (7.5) |  |
| **ALBI Grade** | | | 0.309 |
| I | 32 (60.4) | 36 (67.9) |  |
| II | 19 (35.8) | 17 (32.1) |  |
| III | 2 (3.8) | 0 (0.0) |  |
| **Curative treatment** | | | 0.518 |
| No | 17(32.1) | 13(24.5) |  |
| Yes | 36 (67.9) | 40 (75.5) |  |
| **AFP** (ng/ml) | | | 0.327 |
| <200 | 27(50.9) | 33(62.3) |  |
| ≥200 | 26 (49.1) | 20 (37.7) |  |
| **ALB** (g/L) | 39.91±5.01 | 41.12±4.74 | 0.201 |
| **AST** (U/L) | 71.54±100.75 | 50.70±40.40 | 0.165 |
| **ALT** (U/L) | 57.07±62.60 | 41.84±24.85 | 0.103 |
| **WBC** (10^9^/L) | 6.87±2.97 | 6.10±2.44 | 0.148 |
| **PLT** (10^9^/L) | 175.56±86.38 | 177.32±81.18 | 0.914 |
| **PT** (s) | 12.19±1.38 | 12.09±1.13 | 0.695 |

*ALBI grade: albumin-bilirubin grade，**PT: prothrombin time
