# Supplementary material for: Effect of prior cancer on survival of hepatocellular carcinoma: implications for clinical trial eligibility criteria
Source: BMC Cancer. 2021 Feb 9;21:147. doi: 10.1186/s12885-021-07870-0 (PMC7871582; doi:10.1186/s12885-021-07870-0)
Supplement: Supplementary file 2 — Additional file 2: Supplement Table 2. Independent factors for the survival of HCC patients in 2009–2017 from Our Cancer Center. [file 12885_2021_7870_MOESM2_ESM.docx]

**Supplement Table 2.** Independent factors for the survival of HCC patients in 2009-2017 from Our Cancer Center.

| **Covariates** | **Overall Survival** | | **Progression-free Survival** | |
| --- | --- | --- | --- | --- |
|  | **Univariate**  **analysis** | **Multivariate analysis** | **Univariate**  **analysis** | **Multivariate analysis** |
|  | **P value** | **P value** | **P value** | **P value** |
| **Age** (years) | 0.321 |  | <0.001 | 0.008 |
| **Sex** | 0.438 |  | 0.517 |  |
| **Prior cancer history** | 0.478 | 0.996 | 0.286 | 0.303 |
| **HBV infection** | 0.662 |  | 0.029 | 0.058 |
| **Cirrhosis** | 0.996 |  | 0.335 |  |
| **Tumor size** (mm) | 0.006 | 0.950 | 0.033 | 0.008 |
| **Tumor number** | <0.001 | 0.032 | 0.017 | 0.044 |
| **BCLC stage** | 0.001 | 0.002 | 0.147 |  |
| **ALBI Grade** | <0.001 | 0.174 | 0.843 |  |
| **Curative treatment** | <0.001 | <0.001 | 0.838 |  |
| **AFP** (ng/ml) | <0.001 | <0.001 | 0.059 | 0.186 |
| **ALT** (U/L) | 0.005 | 0.111 | 0.629 |  |
| **ALB** (g/L) | 0.224 |  | 0.307 |  |
| **PT** (s) | 0.035 | 0.833 | 0.819 |  |

*ALBI grade: albumin-bilirubin grade，**PT: prothrombin time
